# 

**Published:** 1907-12-14

**Authors:** 


### THE GRADUATES' MEDICAL SCHOOLS.

### Diary for the week December 16 to 21.

**NATIONAL HOSPITAL FOR THE PARALYSED AND EPILEPTIC, Queen Square, Bloomsbury, W.C.**

**Lectures at 3.30 p.m.**
December 17, Mr. Sargent, **Nerve Anastomosis.**
December 20, Mr. Paton, **Optic Neuritis.**

**NORTH-EAST LONDON POST-GRADUATE COLLEGE, Prince of Wales's Hospital, Tottenham, N.**

**Surgical, Medical and Special Clinics** daily, Saturdays excepted.

**Lectures at 4.30 p.m.**

December 17, Mr. de Prenderville, **The Administration of Anæsthetics.**

**THE POST-GRADUATE COLLEGE, West London Hospital, Hammersmith, W.**

[The Second Winter Session on January 13, 1908.

THE HOSPITAL December 14, 1907.

*Name* ........................................

*Address* ........................................

........................................

This Coupon must accompany manuscript or contributions intended for THE HOSPITAL.

